# Takostubo syndrome combined with ventricular septal perforation: a case report

**DOI:** 10.1186/s12872-022-02834-z

**Published:** 2022-09-08

**Authors:** Kexin Yang, Xinghui LI, Ping Xie, Xiang Zhong, Yifan Zhang, Chenmeng Xiao, Xiaotao Yao, Jia Cai, Meng Lin

**Affiliations:** 1 Lanzhou University, Lanzhou, 730000 Gansu China; 2 Department of Cardiology, Gansu Provincial People’s Hospital, Lanzhou, 730000 Gansu China; 3 Gansu University of Traditional Chinese Medicine, Lanzhou, 730000 Gansu China; 4 Ningxia Medical University, Yinchuan, 750000 Ningxia China

**Keywords:** Takotsubo syndrome, Ventricular septal perforation, Case report

## Abstract

**Background:**

The precise clinical features and etiologic basis of Takotsubo syndrome remain unclear, although an association with emotional or stressful triggers has been recognized. Ventricular septal perforation is a very rare life-threatening complication.

**Case presentation:**

A 77-year-old female patient presented to the hospital with unrelieved chest tightness and shortness of breath. Three months ago, the patient's electrocardiogram revealed ischemic T wave inversion of the anterior wall, along with an increase in myocardial injury markers. There was no evidence of a ventricular septal defect on echocardiography. The patient was admitted to the respiratory department to treat lung lesions. The electrocardiogram demonstrated dynamic changes following admission, and the myocardial markers returned to normal, but the echocardiography revealed a ventricular septal defect. The initial diagnosis was ventricular septal perforation because of myocardial infarction with acute anterior ST-segment elevation. Coronary angiography revealed no abnormalities, but left ventricular angiography revealed an enlarged apex and VSD, with a right ventricular shunt bundle. Later, cardiac MRI revealed an apical ventricular septal defect. Further inquiry of the patient's medical history revealed that her husband died unexpectedly three months ago, and her daughter was seriously injured in a car accident, causing the patient severe emotional distress. Takotsubo syndrome was then determined in conjunction with the patient's medical history, symptoms, signs, and pertinent examinations. Without using a catheter or a surgical procedure, we managed the patient's medical condition. Two weeks later, the patient was discharged with symptoms improved.

**Conclusions:**

Takotsubo syndrome is comparable to an acute myocardial infarction on clinical and electrocardiographic examination in the absence of significant coronary disease. Although ventricular septal perforation is most commonly associated with acute myocardial infarction, it can also happen following Takotsubo syndrome. Takotsubo syndrome complicated by ventricular septal perforation is easily misdiagnosed. The early recognition and management of this condition can avoid or reduce morbidity and mortality.

## Background

Takotsubo syndrome (TTS), apical spherical syndrome, octopus pot cardiomyopathy, and broken heart syndrome are all terms used to describe stress cardiomyopathy [1]. TTS is a reversible form of acute left ventricular failure with a good prognosis that is linked to emotional or stressful stimuli. Ventricular septal perforation (VSP) is a very rare life-threatening complication of TTS that can easily be misdiagnosed as a complication of acute myocardial infarction (MI). We describe the case of TTS complicated by VSP. The importance of early diagnosis of serious complications of TTS and management are highlighted.

## Case presentation

On April 2, 2021, a 77-year-old female patient presented to the hospital with "intermittent chest tightness and shortness of breath for three months, aggravated for three days."

First hospitalization (3 months prior to admission to our hospital): the patient was admitted to a local hospital after experiencing chest tightness and shortness of breath with fatigue because of emotional excitation. The above-mentioned symptoms persisted despite symptomatic and supportive treatment (specifically unknown). Later in the hospital stay, the patient was diagnosed with an acute exacerbation of chronic obstructive pulmonary disease, chronic pulmonary heart disease, grade IV cardiac function, and pleural effusion. After treating the patient with oxygen inhalation, relieving cough and sputum, administering antibiotics, and improving cardiac function, the patient was discharged.

Second hospitalization (3 days prior to transferal to our center): the patient experienced an acute attack of chest tightness and shortness of breath following emotional excitement or even walking 200 m. This symptom may improve with rest, aggravate with lying down, and improve with sitting or standing. Additionally, the patient experienced edema in both lower limbs and was admitted to a nearby county hospital. The echocardiography revealed a septal defect in the left ventricle.

Transferal to our center: after three days in a peripheral center the patient was referred to our institution for further treatment. Further inquiry of the patient's medical history revealed that her husband died unexpectedly three months ago, and her daughter was seriously injured in a car accident, causing the patient severe emotional distress.

Physical examination on admission revealed the following vital signs: body temperature, 36.5 ℃; pulse, 69 times/min; respiratory rate, 22 times/min; blood pressure, 81/58 mmHg; clear consciousness, acute illness face, no cyanosis of lips, no filling of jugular veins, thick breathing sound in both lungs, and moist rales heard in the base of both lungs. There was no precordial bulge, and the apical impulse was located approximately 0.5 cm lateral to the left midclavicular line. A systolic thrill could be palpated between the third and fourth intercostal spaces on the left sternal border. Percussion revealed the left heart border was enlarged. The heart rate had a regular rhythm; a grade 4/6 all-systolic ejection murmur was heard between the third and fourth intercostal spaces on the left sternal border, and a grade 3/6 systolic blowing murmur was heard in the tricuspid area. Cardiac examination findings highly indicated that there was a suspected ventricular septal defect (VSD). The abdomen of the patient was soft; the liver and spleen were not palpable beneath the ribs, and the patient's lower limbs were mildly edematous.

**Fig. 1:**
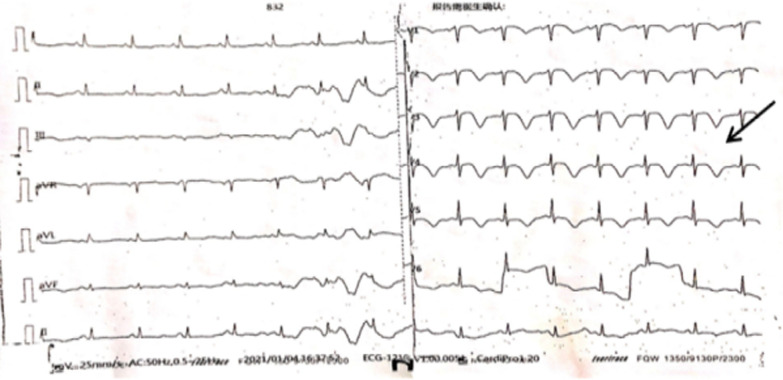
The patient’s ECG results three months before admission (2021-01-04) (limb lead low voltage; extensive T-wave inversion chest lead)

A review of the self-copied medical record indicated following findings during the hospitalization three months prior to the referral to our institution: The Electrocardiogram (ECG) determined the following: limb lead low voltage, T wave flat, and V1-V6 lead T wave inversion (Fig. 1); The echocardiography revealed the following: dilatation of the right heart (RA Diameter = 50 mm, RV Diameter = 30 mm), pulmonary hypertension (sPAP = 54 mmHg), aortic sclerosis, mitral regurgitation (Grade 1), tricuspid regurgitation (Grade 3), normal left ventricular systolic function (EF 65%), and bilateral pleural effusion; The Chest CT scan showed scattered exudative foci in both lungs possible pulmonary edema associated with infection, localized honeycomb changes in both lungs' inferior lobes, possible localized pulmonary interstitial fibrosis, subsegmental atelectasis in the inferior lingual segment of the superior lobe of the left lung, bilateral pleural effusion resulting in pulmonary atelectasis in the lungs’ adjacent lower lobes, a small amount of pericardial effusion, and a localized thickening and adhesion of bilateral pleura.

**Fig. 2:**
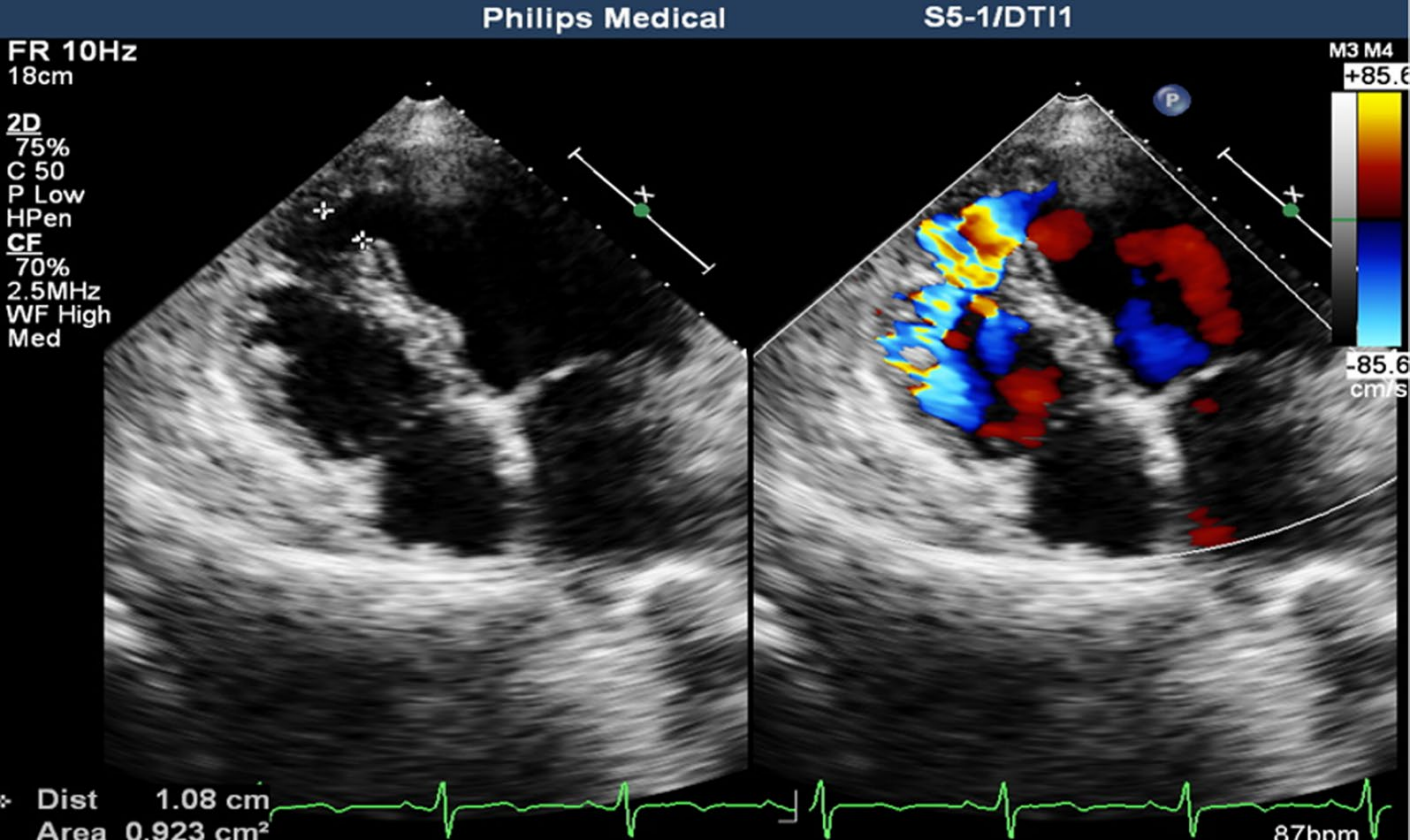
The patient’s cardiac ultrasound results after admission (apical ventricular septal had a defect of 10.8 × 4.1 mm; left-to-right shunt of colored blood flow)

Second hospitalization (3 days prior to transferal to our center): The hospital’s echocardiography determined the following: dilatation of the right heart (RA Diameter = 52 mm, RV Diameter = 31 mm), enlargement of the left heart (LA Diameter = 48 mm, LVIDd / LVIDs = 54 / 33 mm), apical ventricular septal defect (10.8 X 4.1 mm), a left-to-right shunt at ventricular level, a mild mitral regurgitation, a severe tricuspid regurgitation, increased pulmonary artery pressure (sPAP = 69 mmHg), and normal left ventricular systolic function (EF 70%) (Fig. 2). The results of echocardiography confirmed the suspected diagnosis of VSD at admission.

**Fig. 3:**
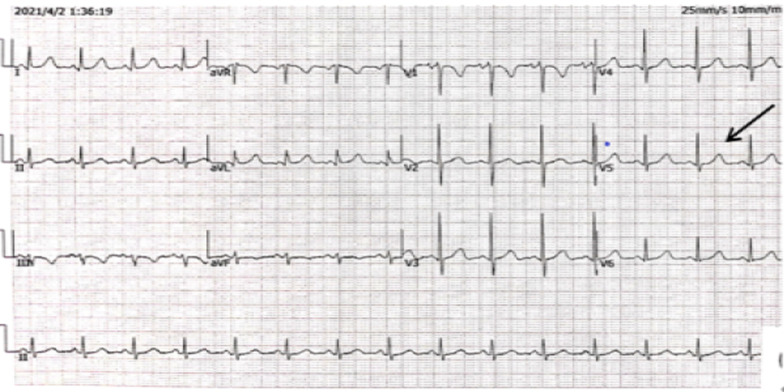
The patient’s ECG results upon admission (2021-04-02) (disappearance of limb lead low voltage; T wave inversion in chest lead)

After admission to our hospital, no significant abnormality was noted in the ECG results; the low voltage in the limb leads had vanished, and the T waves in leads V1-V6 were upright (Fig. 3). The results of laboratory examinations were as follows: CKMB, 1.1 ng/ml; MYO, 71.0 ng/ml; TNI, < 0.05 ng/ml; BNP, 590 pg/ml; DDIM, 759 ng/ml. The arterial blood gas provided the following indicators: pH, 7.46; SaO_2_, 91.1%; PaO_2_, 60.90 mmHg; PaCO_2_, 23.00 mmHg; cLAC, 1.1 mmol/L; cHCO_3_-, 19.40 mmol/L. The patient's hematuria and stool routines, liver and kidney functions, blood lipid, blood sugar, ion, and coagulation function were not abnormal.

The initial diagnoses were as follows: (1) anterior myocardial infarction? complications of the ventricular septal defect; (2) stable chronic obstructive pulmonary disease, chronic pulmonary heart disease (decompensated).

**Fig. 4:**
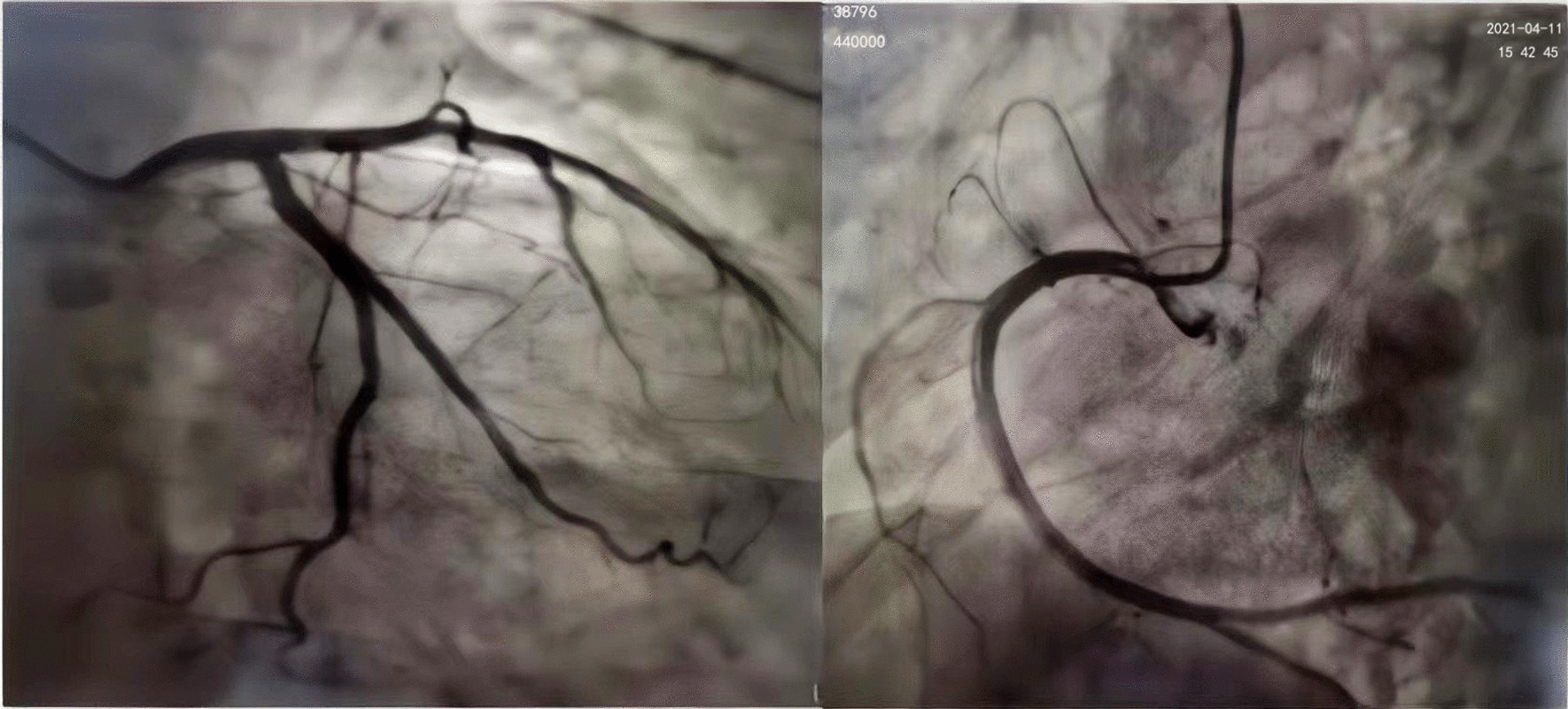
The patient’s coronary angiography results after admission (no obvious abnormality in the left and right coronary arteries)

**Fig. 5:**
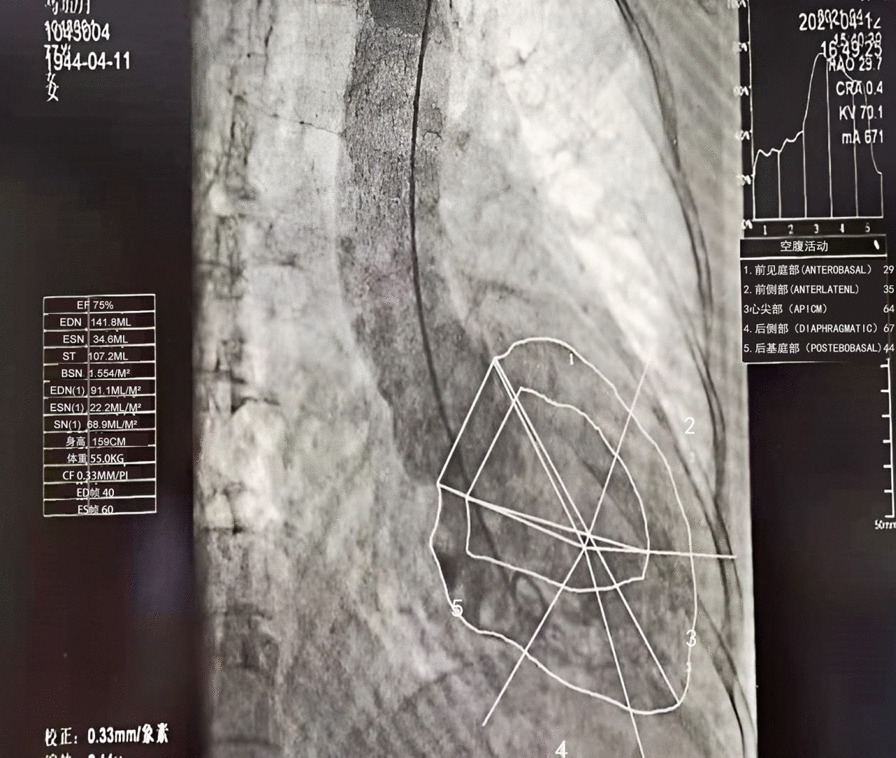
The patient’s Left ventriculography results (local expansion of cardiac apex; mild abnormal wall motion)

**Fig. 6:**
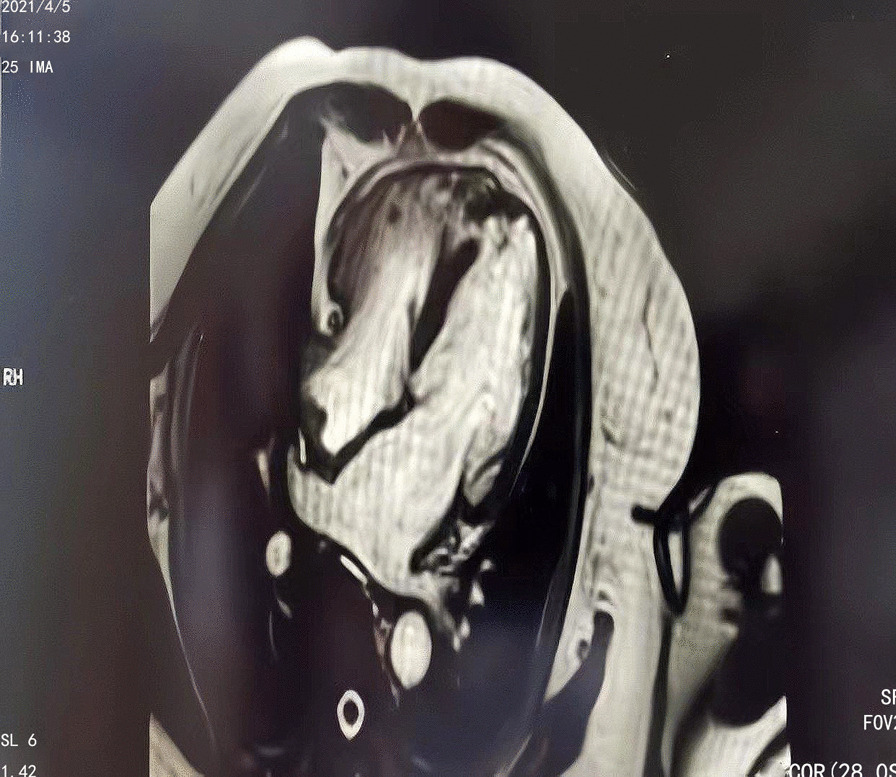
The patient’s LGE images results (apical ventricular septal defect of 7.3 × 3.8 mm; mild abnormal wall motion; localized fibrosis; intimal edema)

The patient was then subjected to coronary angiography, which revealed no significant abnormalities (Fig. 4), but left ventricular angiography revealed mild apical dilatation, mild abnormal wall motion (Fig. 5) and VSD (8.7 X 4 mm), with a right ventricular shunt bundle. The cardiac MRI revealed a VSD (7.3 X 3.8 mm), a mild mitral insufficiency, a severe tricuspid insufficiency, a mild aortic insufficiency, cardiac enlargement, predominantly left atrioventricular enlargement, and pulmonary hypertension. Additionally, the late gadolinium enhancement (LGE) images revealed intimal edema and local fibrosis (Fig. 6).

Stress cardiomyopathy, secondary VSP, left ventricular enlargement, and cardiac function grade IV were all the amended diagnoses.

VSD can be treated percutaneously with symmetrical umbrella-type occluder devices under X-ray guidance. Considering the defect of the patient was located at the apex of the heart, the diameter was relatively large, it was considered too difficult and risky to perform interventional closure treatment. Post- consultation with a cardiac surgeon, it was determined that the patient was elderly and had a history of poor pulmonary function, and that surgical treatment would be feasible if the family were willing to accept the risk. However, the patient and her family vehemently refused surgery. Psychological suggestive, low-flow oxygen inhalation, aspirin 0.1 g QD, rosuvastatin 10 mg QN, furosemide 20 mg QD, spironolactone 20 mg QD, and micropump injection of recombinant human brain natriuretic peptide 0.01 ug/kg/min were all used to treat.

Two weeks later, the patient was discharged due to improved symptoms of chest tightness and shortness of breath and a stable condition assessment. Vital signs at discharge were: body temperature, 36.4 ℃; pulse, 87 times/min; respiratory rate, 20 times/min; blood pressure, 114/78 mmHg. She was instructed to continue taking furosemide 20 mg QD, spironolactone 20 mg QD, sacubitril-valsartan sodium tablets 50 mg BID, and metoprolol tartrate 25 mg BID via oral administration. During the three follow-up visits outside the hospital, the patient experienced no significant discomfort in her daily activities.

## Discussion and conclusions

Increased mental or physical stress, as seen in the present case, typically occur prior to the onset of TTS, which is characterized by transient apical ventricular wall motion abnormalities and balloon-like changes. TTS is comparable to an acute coronary syndrome, except that there is no evidence of obstructive coronary heart disease or acute plaque rupture on angiography [1]. Chest tightness and shortness of breath are the primary symptoms; there are no typical chest pain symptoms. Our patient, an elderly woman presented with a history of great traumatic stress and nonspecific chest tightness and shortness of breath. Physical examination revealed an enlarged left heart border and a grade 3/4 systolic murmur on the left sternal border. The ECG in such patients can show dynamic changes, and myocardial injury markers may be increased. Clinically acute myocardial infarction was suspected, but coronary angiography clearly ruled out this diagnosis. Combined with the echocardiography findings, cardiac MRI and left ventricular angiography, TTS was confirmed. After psychological suggestive and symptomatic treatment, the symptoms could be relieved, further verifying the diagnosis of TTS.

TTS patients are 90% female, average 67–70 years old, and approximately 80% are over 50 years old. Sympathetic stimulation plays a critical role in pathogenesis, but the precise mechanism remains unknown [2]. Studies have shown that estrogen reduces plasma catecholamine levels by activating the β2AR-Gi protein signaling pathway and induces endothelial nitric oxide synthase synthesis to protect the myocardium from damage, while estrogen also modulates myocardial catecholamine sensitivity. Thus, postmenopausal women's myocardium is susceptible to catecholamine toxicity during times of stress [3]. The patient is an elderly woman who lives in a remote rural area and has been experiencing significant grief for the last three months because of a sudden family accident. In this case, emotional stress results in a rapid release of catecholamines, thereby increasing epinephrine and norepinephrine levels in the circulatory system and causing myocardial toxicity [4].

Acute heart failure, mitral regurgitation, and cardiogenic shock are the most common complications of TTS; atrioventricular block, left ventricular thrombosis, VSP, and cardiac rupture are uncommon [5]. The incidence of ventricular free wall rupture has been reported to be 0.5%, and fewer than 20 cases of VSP have been reported [6]. Advanced age, female gender, hypertension, and persistent ST-segment elevation are all risk factors for ventricular wall perforation and rupture. Additionally, elevated TNI and CK levels, increased left ventricular wall internal pressure and stress are clinical predictors for ventricular wall rupture in TTS patients [6, 7]. TTS is associated with a favorable prognosis, and conservative treatment can improve left ventricular function in most patients. However, ventricular wall rupture, particularly in the free wall, can result in death in some cases. In this case, VSP occurred in the cardiac apex without fatal consequences, owing to the insignificant hemodynamic effect of apical perforation.

TTS is an underdiagnosed condition that currently lacks a standardized treatment. Most TTS patients are treated conservatively, with an emphasis on relieving physical or emotional stressors that contribute to the disease's onset. Complications such as acute heart failure and cardiogenic shock, on the other hand, may necessitate the use of mechanical adjuvant therapy such as IABP and ECMO [8]. In terms of drugs, β-receptor blockers are recommended to prevent progress and recurrence. Angiotensin converting enzyme inhibitors [8] (ACEI) or angiotensin receptor blockers (ARB) are related to improving survival. Some studies have determined that β-receptor blockers therapy is associated with a low incidence of cardiac rupture [9]. Following in-hospital treatment, this patient's condition improved because of psychological hints, mental reassurance, cardiac load reduction, and infusion of synthetic brain natriuretic peptide, among other interventions. The patient's condition stabilized following out-of-hospital administration of regular oral diuretics, sacubitril, valsartan, and metoprolol.

## Data Availability

All data generated or analysed during this study are included in this published article.

## Abbreviations

TTS: Takotsubo syndrome
VSP: Ventricular septal perforation
VSD: Electrocardiogram
MI: Myocardial infarction
ECG: Electrocardiogram
UCG: Ultrasound Cardiogram
CAG: Coronary angiography
ACEI: Angiotensin converting enzyme inhibitor
CK-MB: Creatine Kinase
MYO: Myoglobin
TNI: Troponin
BNP: Brain Natriuretic Peptide
